# Systematic literature review of quality‐of‐life questionnaires in Waldenström macroglobulinaemia—need for a disease‐specific tool

**DOI:** 10.1002/jha2.668

**Published:** 2023-03-21

**Authors:** Sotirios Bristogiannis, Jahanzaib Khwaja, Yadanar Lwin, Encarl Uppal, Shirley D'Sa, Charalampia Kyriakou

**Affiliations:** ^1^ Department of Haematology and Bone Marrow Transplantation Unit Evangelismos Hospital Athens Greece; ^2^ Department of Haematology NHS University College London Hospital London UK; ^3^ Department of Haematology Nottingham University Hospitals NHS Trust Nottingham UK; ^4^ ULCH Centre for Waldenström's and Related Conditions, Cancer Division UCLH NHS Foundation Trust London UK

**Keywords:** quality of life, Waldenström's macroglobulinaemia

1

Waldenström macroglobulinaemia (WM) is a rare B‐cell lymphoma associated with monoclonal immunoglobulin‐M (IgM). It can remain asymptomatic for years but ultimately a proportion will develop symptomatic manifestations requiring treatment. Uniquely, these may be related to lymphoma infiltration or the monoclonal protein itself causing associated phenomena (neuropathy, autoimmune complications and cryoglobulinaemia). WM is an incurable but treatment‐responsive disease with multiple treatment options that have advanced survival. Treatments include chemoimmunotherapy and targeted agents, of fixed or continuous duration [1, 2]. As more effective therapies develop, disease and therapy‐related quality of life (QoL) is increasingly recognised as an important endpoint. Although patients can engage in and evaluate the effects of the disease on their QoL [3], no WM‐specific validated QoL assessment tool exists. Therefore, we conducted a literature review of the currently used QoL tools in WM.

A systematic search of PubMed, Medline and EMBASE databases was conducted for QoL Questionnaires (QLQs) applied in literature for patients with WM up to December 2022. Keywords used were ‘Waldenström Macroglobulinaemia’ OR ‘Lymphoplasmacytic Lymphoma’ AND ‘Quality of Life’ OR ‘Patient Reported Outcomes’. Supplementary search was performed using common complications of the disease, for example, polyneuropathy, established treatment regimens and quality of life as keywords.

Sixty‐six publications were identified and screened for relevance to the study by two independent investigators (Figure 1). The following data were extracted from each study: number of patients, study design (observational or interventional study), QLQs used, evidence for psychometric validation and health domains assessed. Next, the evidence for validation of these QLQs in the context of WM was evaluated according to Consensus‐based Standards for the selection of health Measurement Instruments (COSMIN) guidelines (Supplementary Informations S1 and S2). The quality of this proof was graded as per van Tulder et al. standards (Supplementary Information S3) [4].

**FIGURE 1:**
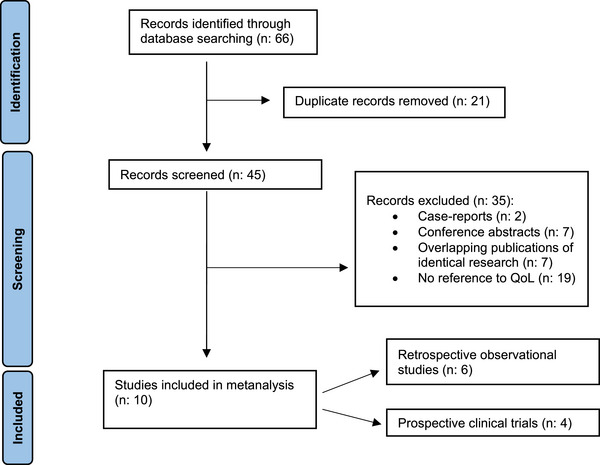
Meta‐analysis flowchart.

Of the 10 studies identified, 14 non‐mutually exclusive questionnaires were applied, often in combination, to report QoL: European Organisation for the Research and Treatment of Cancer Quality of Life Questionnaire‐Cancer30 (EORTC QLQ‐C30), five level EuroQol five‐dimensional questionnaire (EQ‐5D‐5L), Functional Assessment of Cancer Therapy‐General (FACT‐G), Functional Assessment of Chronic Illness Therapy‐Fatigue (FACIT‐F), FACT‐Anaemia (FACT‐An), FACT‐Gynecologic Oncology Group‐Neurotoxicity (FACT‐GOG NTx), FACT‐Lymphoma (FACT‐Lym), 36‐Item Short Form Survey (SF‐36), Hospital Anxiety and Depression Scale (HADS), Depression Anxiety Stress Scales‐21 (DASS‐21), Quality of Life in Cancer Survivors (QLACS), Impact of Event Scale‐6 (IES‐6), Fatigue Severity Scale (FSS) and Impact on Participation and Autonomy questionnaire (IPA). All studies were dated from 2016. Most QLQs were originally validated in cancer cohorts (9/17; 53%) and one in lymphoma patients (FACT‐Lym). These questionnaires addressed several physical and mental issues of patients with WM at variable levels (Table 1). Table 1 summarises the content validity of the utilised QLQs (colour‐coded according to the directness of assessment).

**TABLE 1:** Patient‐reported HRQoL indicators in health‐related quality of life questionnaires used in literature for patients with Waldenström's macroglobulinaemia.

	*Health limitations*					*Symptom burden*							
	Energy	Physical ability	Cognition	Family role	Social role	Mental health	Insomnia	Pain	Constipation	Dyspnea	Appetite loss	Diarrhea	Nausea
**EORTC QLQ‐C30**													
**EQ‐5D‐5L**													
**FACT‐G**													
**FACIT‐F**													
**FACT‐An**													
**FACT‐GOG**													
**FACT‐Lym**													
**SF‐36**													
**HADS**													
**DASS‐21**													
**QLACS**													
**IES‐6**													
**FSS**													
**IPA**													

*Note*: Green: directly assessed/orange: indirectly concluded/red: not assessed.

Abbreviations: EORTC QLQ‐C30, European Organisation for the Research and Treatment of Cancer Quality of Life Questionnaire‐Cancer30; EQ‐5D‐5L, five level EuroQol five‐dimensional questionnaire; FACT‐G, Functional Assessment of Cancer Therapy‐General; FACIT‐F, Functional Assessment of Chronic Illness Therapy‐Fatigue; FACT‐An, Functional Assessment of Cancer Therapy‐Anaemia; FACT‐GOG, Functional Assessment of Cancer Therapy/ Gynecologic Oncology Group‐Neurotoxicity; FACT‐Lym, Functional Assessment of Cancer Therapy‐ Lymphoma; SF‐36, 36‐Item Short Form Survey; HADS, Hospital Anxiety and Depression Scale; DASS‐21, Depression Anxiety Stress Scales‐21; QLACS, Quality of Life in Cancer Survivors; IES‐6, Impact of Event Scale; FSS, Fatigue Severity Scale; IPA, Impact on Participation and Autonomy questionnaire.

EQ‐5D‐5L was the most commonly (8/17; 47%) employed questionnaire [5, 6, 7, 8, 9, 10, 11, 12, 13, 14]. FACT‐GOG assessed QOL burden when the disease was complicated by peripheral neuropathy.

There was no evidence for validation for WM of any of these QLQs against any COSMIN psychometric properties except for content validity (S1). In psychometrics, QLQs’ content validity is assessed by consulting patients and physicians (face‐validity studies). In the absence of face‐validity studies, we evaluated the content validity subjectively by examining whether QLQs referred to disease health effects as acknowledged by patients in open‐question surveys in the literatures [12]. EORTC QLQ‐C30 was the only QLQ that adequately addressed all these effects. Selective QLQs delved further into specific aspects including emotional burden (HADS and DASS‐21)

Despite their lack of specificity for WM, these QLQs have led to useful observations about the QoL of these patients.

Traditionally, the QoL of patients with WM has been related to age and comorbidities, [6] but the disease itself may adversely affect patients’ mental health. Indeed, newly diagnosed patients report post‐traumatic stress, which diminishes gradually provided no treatment is required [13]. Once, though, the disease becomes symptomatic, both the disease itself and the treatment may generate troublesome symptoms such as digestive upset, dyspnea and pain that limit patients’ physical, cognitive function, social and family role and disturb their sleep quality [11, 12]. QoL burden is more pronounced in patients with disease‐associated polyneuropathy [6, 7, 8].

Four clinical trials reporting QoL data were identified in the literature and all employed a combination of QLQs [5, 9, 10, 14]. These include a small number of research participants (415 patients altogether) reflecting the disease's rarity. Bruton tyrosine kinase inhibitors appear more tolerable than traditional chemoimmunotherapy regimens, even for pre‐treated patients. In the phase III trial iNNOVATE, comparing ibrutinib–rituximab to placebo–rituximab, ibrutinib–rituximab improved patients’ QoL within 1–2 months, in parallel with the decrease in IgM component [5]. In the phase III ASPEN trial comparing zanubrutinib to ibrutinib, zanabrutinib showed more favourable QoL outcomes thanks to its more manageable toxicity profile [9]. In the HOVON124 phase I/II study in relapsed WM, ixazomib resulted in the improvement of all functional aspects except physical and cognitive function without deteriorating neuropathy [14]. Last, the phase III CHRONOS‐3 trial failed to show any QoL benefit for patients with non‐Hodgkin lymphoma including 38 patients with WM that received copanlisib in addition to rituximab versus placebo [10].

WM is a rare disease with a broad spectrum of presentations varying from the indolent to affecting various organ systems, often in parallel. Targeted treatments promise effective, long‐term control of the disease. It is anticipated that these would benefit the QoL of patients. We demonstrate here that limited data on this aspect are available in the literature. QoL outcomes have been only lately been reported in the literature for WM since effective treatments have improved survival outcomes, and such data have been mandated by medicines agencies [15]. Late effects of new treatments, particularly continuous maintenance therapy, are important to establish when considering the long‐term benefit. QLQs employed in patients with WM are adopted from related conditions and are often used in combination. Our analysis failed to validate any of these tools against all COSMIN standards and only EORTC QLQ‐C30 had solely acceptable content validity for WM. Thus, there is a need to develop a new QLQ designed specifically to address adequately the QoL issues of WM patients, The core QLQ should be applicable to all patients with WM. Additional QoL aspects relevant to those with IgM‐associated disorders (e.g., with peripheral neuropathy) could be investigated further using available databases of questions addressing specific QoL issues (a.k.a. Item Libraries). It is anticipated that such QLQs would enable physicians to adjust the treatment according to the QoL impact. It is imperative that researchers provide essential reliable long‐term QoL data for future drug approval.

## AUTHOR CONTRIBUTIONS

Sotirios Bristogiannis designed, performed the research, analysed the data and wrote the paper. Charalampia Kyriakou contributed to the design of the research, the analysis of the data and the writing of the paper. Jahanzaib Khwaja contributed to the data analysis and paper writing. Yadanar Lwin contributed to the design and implementation of the research. Shirley D'Sa contributed to the design of the research and the writing of the paper.

## CONFLICT OF INTEREST STATEMENT

Shirley D'Sa reports speaker fees and self‐directed research funding from Janssen, BeiGene and Sanofi.

The rest of the authors declare no competing financial or non‐financial interests.

## ETHICS STATEMENT

The authors have confirmed ethical approval statement is not needed for this submission.

## CLINICAL TRIAL REGISTRATION

The authors have confirmed clinical trial registration is not needed for this submission.

## PATIENT CONSENT STATEMENT

The authors have confirmed patient consent statement is not needed for this submission.

## Supporting information

Supporting Information

## Data Availability

None.
