# Supplementary material for: Systematic literature review of quality‐of‐life questionnaires in Waldenström macroglobulinaemia—need for a disease‐specific tool
Source: EJHaem. 2023 Mar 21;4(2):555–8. doi: 10.1002/jha2.668 (PMC10188456; doi:10.1002/jha2.668)
Supplement: Supplementary file 1 — Supporting Information [file JHA2-4-555-s001.docx]

| Domain | Measurement/Psychometric Properties | | Definition |
| --- | --- | --- | --- |
| Reliability |  | | The degree to which the Quality of Life evaluation using the assessed QLQ is free from measurement error. |
|  | Reliability (extended definition) | | The extent to which the assessed QLQ scores for patients who have not changed are the same for repeated measurements under different conditions: e.g. using different sets of questions referring to Quality of Life domains (‘Internal Consistency’); over time (‘test-retest’); by different person on the same occasion (‘inter-rater’) or by the same persons on different occasions (‘intra-rater’) |
|  | Internal Consistency | | The degree of the interrelatedness among various questions referring to the Quality of Life of the population of interest. |
|  | Measurement Error | | The systematic and random error of a patient’s score that is not attributed to true changes in the quality of life. |
| Validity |  | | The degree to which a QLQ* measures the Quality of Life of the population of interest. |
|  | Content Validity | | The degree to which the content of a QLQ* is an adequate reflection of the Quality of Life of the population of interest. |
|  | Structural Validity |  | The degree to which the scores of a QLQ* are an adequate reflection of the dimensionality of the Quality of Life of the population of interest. |
|  |  | Hypothesis Testing | Idem construct validity. |
|  |  | Criterion Validity | The degree to which the scores of the assessed QLQ are an adequate reflection of a ‘gold standard’ QLQ for the population of interest. |
| Responsiveness | Responsiveness | | The ability of a QLQ to detect change over time in the Quality of Life of the population of interest. |

**S1: *Adaptation* of the COSMIN definitions for domains, measurement properties and aspects of measurement properties *in the context of Quality of Life Questionnaires*.** *† The word ‘true’ must be seen in the context of the* Intraclass Correlation Coefficient (*CTT) [see S2], which states that any observation is composed of two components – a true score and error associated with the observation. ‘True’ is the average score that would be obtained if the scale were given an infinite number of times. It refers only to the consistency of the score, and not to its accuracy *QLQ: Quality-of-Life Questionnaire*

| *Measurement Property* | *Rating* | *Criteria* |
| --- | --- | --- |
| *Reliability* | *+* | ICC or weighted Kappa ≥ 0.70 |
|  | *?* | ICC or weighted Kappa not reported |
|  | *-* | ICC or weighted Kappa < 0.70 |
| Internal consistency | *+* | At least low evidence for sufficient structural validity AND Cronbach's alpha(s) ≥ 0.70 for each unidimensional scale or subscale |
|  | *?* | Criteria for “At least low evidence for sufficient structural validity” not met |
|  | *-* | At least low evidence for sufficient structural validity AND Cronbach’s alpha(s) < 0.70 for each unidimensional scale or subscale |
| Measurement error | *+* | SDC or LoA < MIC |
|  | *?* | MIC not defined |
|  | *-* | SDC or LoA > MIC |
| *Content validity* | *+* | All items refer to relevant aspects of the construct to be measured AND are relevant for the target population AND are relevant for the purpose of the measurement instrument AND together comprehensively reflect the construct to be measured |
|  | *?* | Not all information for ‘+’ reported |
|  | *-* | Criteria for ‘+’ not met |
| Structural validity | *+* | **CTT:**  CFA: CFI or TLI or comparable measure >0.95 OR RMSEA <0.06 OR SRMR <0.08  **IRT/Rasch**: No violation of unidimensionality: CFI or TLI or comparable measure >0.95 OR RMSEA <0.06 OR SRMR <0.08 *AND* no violation of local independence: residual correlations among the items after controlling for the dominant factor < 0.20 OR Q3's < 0.37 *AND* no violation of monotonicity: adequate looking graphs OR item scalability >0.30 *AND* adequate model fit: IRT: χ2 >0.01 Rasch: infit and outfit mean squares ≥ 0.5 and ≤ 1.5 OR Z‐ standardized values > ‐2 and <2 |
|  | *?* | CTT: Not all information for ‘+’ reported IRT/Rasch: Model fit not reported |
|  | *-* | Criteria for ‘+’ not met |
| Hypotheses testing for construct validity | *+* | The result is in accordance with the hypothesis |
|  | *?* | No hypothesis defined (by the review team) |
|  | *-* | The result is not in accordance with the hypothesis |
| Criterion validity | *+* | Correlation with gold standard ≥ 0.70 OR AUC ≥ 0.70 |
|  | *?* | Not all information for ‘+’ reported |
|  | *-* | Correlation with gold standard < 0.70 OR AUC < 0.70 |
| Responsiveness | *+* | The result is in accordance with the hypothesis OR AUC ≥ 0.70 |
|  | *?* | No hypothesis defined (by the review team) |
|  | *-* | The result is not in accordance with the hypothesis7 OR AUC < 0.70 |

**S2:COSMIN criteria for evaluation of the measurement /psychometric properties of a Patient-Reported Outcome Measure (PROM) or here a Quality-of-Life Questionnaire).** AUC: Area Under the Curve, CFA: Confirmatory Factor Analysis, CFI: Comparative Fit Index, CTT: Classical Test Theory, DIF: Differential Item Functioning, ICC: Intraclass Correlation Coefficient, IRT: Item Response Theory, LoA: Limits of Agreement, MIC: Minimal Important Change, RMSEA: Root Mean Square Error of Approximation, SEM: Standard Error of Measurement, SDC: Smallest Detectable Change, SRMR: Standardized Root Mean Residuals, TLI = Tucker‐Lewis index. Refer to (Prinsen et al., Qual Life Res., 2018) for further information.

| Level | Rating | Criteria |
| --- | --- | --- |
| Strong | +++ or --- | Consistent findings in multiple studies of good methodological quality OR in one study of excellent methodological quality. |
| Moderate | ++ or -- | Consistent findings in multiple studies of fair methodological quality OR in one study of good methodological quality. |
| Limited | + or - | One study of fair methodological quality. |
| Conflicting | +/- | Conflicting findings |
| Unknown | ? | Only studies of poor methodological quality. |

S3: COSMIN level of evidence for evaluation of measurement properties
